# Short-Term Resveratrol Exposure Causes *In Vitro* and *In Vivo* Growth Inhibition and Apoptosis of Bladder Cancer Cells

**DOI:** 10.1371/journal.pone.0089806

**Published:** 2014-02-25

**Authors:** Mo-Li Wu, Hong Li, Li-Jun Yu, Xiao-Yan Chen, Qing-You Kong, Xue Song, Xiao-Hong Shu, Jia Liu

**Affiliations:** Liaoning Laboratory of Cancer Genetics and Epigenomics and Department of Cell Biology, College of Basic Medical Sciences, Dalian Medical University, Dalian, China; Columbia University, United States of America

## Abstract

Conventional adjuvant chemotherapies for bladder transitional cell carcinomas (TCCs) may cause strong systemic toxicity and local irritation. Non-toxic resveratrol inhibits TCC cell growth but its feasibility in clinical management of TCCs remains obscure. This study aimed to evaluate the safety and anti-TCC efficacy of resveratrol, using the experimental models closer to the clinical treatment condition. Human TCC EJ cells were exposed to 100 µM, 150 µM and 200 µM resveratrol respectively for 1 hour and 2 hours to mimic intravesical drug instillation and the cell responses were analyzed by multiple experimental approaches. An orthotopic TCC nude mouse model was established by injecting EJ cells into the sub-urothelial layer and used for short-term intravesical resveratrol instillation. The safety of resveratrol instillation was evaluated and compared with that of MCC. The results revealed that 2 h 150 µM or 200 µM resveratrol treatment leaded to remarkable S phase arrest and apoptosis at 72 h time-point, accompanied with attenuated phosphorylation, nuclear translocation and transcription of STAT3, down-regulation of STAT3 downstream genes (survivin, cyclinD1, c-Myc and VEGF) and nuclear translocations of Sirt1 and p53. The importance of STAT3 signaling in cell growth was confirmed by treating EJ cells with JAK2 inhibitor tyrphostin AG490. The efficacy and safety of resveratrol instillation were proved by the findings from nude mouse orthotopic xenograft models, because this treatment caused growth suppression, distinctive apoptosis and STAT3 inactivation of the transplanted tumors without affecting normal urothelium. Our results thus suggest for the first time the practical values of resveratrol as a safe and effective agent in the post-operative treatment of TCCs.

## Introduction

Bladder cancer is the commonest malignancy of the urinary tract, of which 90% is transitional cell carcinoma (TCC). Transurethral resection followed by intravesical chemotherapy is the standard care of TCC patients [1]. Recurrence is the leading risk of TCC patients because of the difficulty to radically remove the aggressive tumors [2]. Consequently, adjuvant intravesical chemotherapies become the major approaches to prevent TCC relapse. Bacillus Calmette-Guerin, interferon-α, cisplatin, mitomycin C (MMC) and their combinations are conventionally used in clinical practice, while their efficacies are variable [3], [4] and usually cause strong systemic toxicity and local complications such as hemorrhagic cystitis [2]. It is therefore in urgent need to explore lesser toxic and more effective approach for better management of TCCs.

Resveratrol has been regarded as a non-toxic polyphenolic compound that found in grapes, berries, peanuts and red wine [5]. A body of evidence shows that resveratrol is able to inhibit the growth of many cancers such as leukemia, breast cancer and primary brain tumors [6]–[8]. In the case of bladder cancers, resveratrol effectively decreases cell viability and induces apoptosis of human and murine bladder cancer cells [9]–[12]. Nevertheless, the practical value of resveratrol in anti-TCC therapy has not been addressed by the use of more clinically relevant experimental model(s) and in the way of local drug administration. In the current study, human TCC cell line, EJ [13], was treated in short term by resveratrol to mimic clinical drug instillation [14]. The cellular and molecular responses of EJ cells to the treatment were analyzed by multiple approaches. Meanwhile, an orthotopic TCC nude mouse model was established by injecting EJ cells into the sub-urothelial layer and treated by resveratrol in the manner similar with intravesical drug instillation [15]. The cellular and molecular responses to those treatments were evaluated thereafter.

## Materials and Methods

### Cell Culture and Treatments

Human TCC EJ cells [13] were cultured in Dulbecco’s modified Eagle’s essential medium (DMEM) containing 10% fetal bovine serum (Gibco Life Science, Grand Island, NY, USA) under 37°C and 5% CO_2_ conditions. The cells (5×10^4^/ml) were plated to culture dishes (NUNC, Denmark) and incubated for 24 h before the experiments.

**Figure 1:**
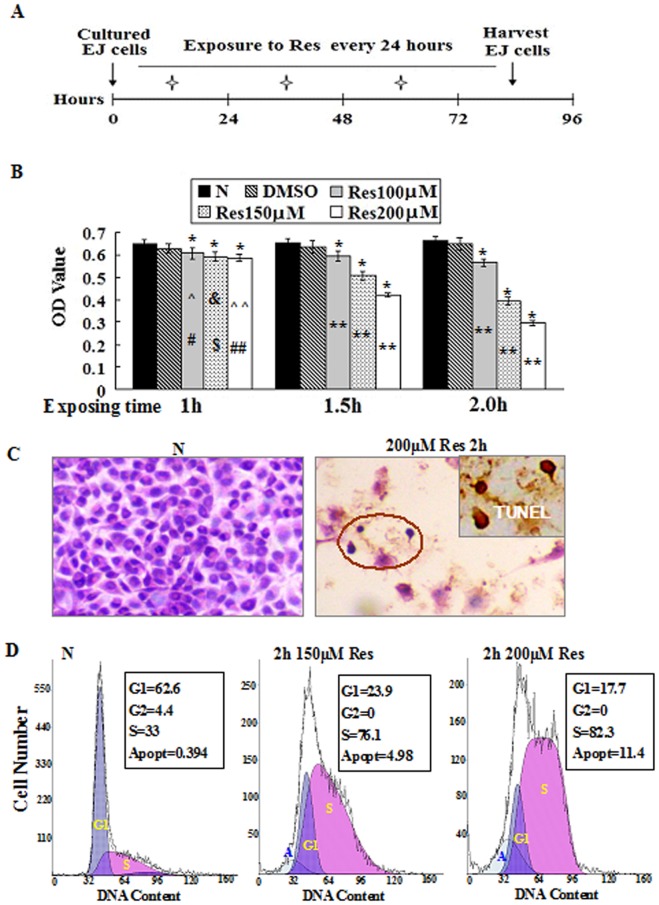
Short-term resveratrol treatments reduced cell viability and the growth of EJ bladder cancer cells, and caused apoptosis of EJ cells in vitro. A. Schematic diagram of short-term in vitro resveratrol treatment. B. MTT assay performed at 72 h after the treatments showed that short-term resveratrol exposure decreased cell viability and suppressed EJ cell growth in dose- and time-dependent manner. *, compared with N group, *P*<0.01; **, compared among 100 µM, 150 µM and 200 µM Res-treated groups at 1.5 h and 2.0 h, *P*<0.01; #, compared with 1 h 150 µM Res treatment, *P*>0.05; $, compared with 1 h 200 µM Res treatment, *P*>0.05; ##, compared with 1 h 100 µM Res treatment, *P*<0.05. ^∧^, compared among 1.0 h, 1.5 h or 2.0 h 100 µM Res-treated groups, *P*<0.01; &, compared among 1.0 h, 1.5 h or 2.0 h 150 µM Res-treated groups, *P*<0.01; ^∧∧^, compared among 1.0 h, 1.5 h or 2.0 h 200 µM Res-treated groups, *P*<0.01. C. H&E staining and TUNEL assay (inset of the cycled region) performed on 2 h 200 µM resveratrol treated-EJ cells at 72 h. D. Flow cytometry was performed to determine cell cycle distribution and apoptosis in EJ cells after short-term resveratrol treatments. 150 µM and 200 µM resveratrol treatments for 2 h could both arrest cell cycle at S phase and increase apoptotic fractions at 72 h in dose-dependent fashion.

Resveratrol (Res; Sigma Chemical, Inc, St. Louis, MO) was dissolved in dimethylsulfoxide (DMSO; Sigma) and diluted with culture medium to the working concentrations just before use. The cells under normal culture condition, treated by 0.2% DMSO and exposed to 100 µM Res for 48 h were used as normal, background and efficacy controls, respectively. As shown in the diagram (Figure 1A), EJ cells were treated by 100 µM, 150 µM or 200 µM Res for 1 h, 1.5 h or 2 h in 24 h intervals. After 1 h and 2 h treatments, Res containing media were replaced with normal medium upon 3 washes. Therefore, EJ cells were exposed to different concentrations of Res for 3 times (once a day) during the 72 h experiment (Figure 1A). Cell numbers and viabilities were checked in 12 h intervals. The cell-bearing coverslips were fixed in cold acetone or 4% paraformaldehyde (pH 7.4) for morphological and immunocytochemical examinations. The experimental groups were set in triplicate and the experiments were repeated for three times to establish confidential conclusion.

### Cell Proliferation and Death Assays

The effects of resveratrol on cell proliferation were determined by 3-[4,5-Dimethylthiazol-2-yl]-2,5-diphenyl-tetrazolium bromide (MTT) assay described elsewhere [8]. The results were shown as percentage of cell viability (OD of the experiment samples/OD of the control) or OD values. Terminal deoxynucleotide transferase (TdT)–mediated dUTP-biotin nick-end labeling (TUNEL) assay was employed to detect apoptotic cells according to producer’s instructions (Promega Corporation, USA). Haematoxylin and eosin (H/E) staining was performed to observe the morphological changes of EJ cells after treatment.

### Flow Cytometry

The harvested cells of the experimental groups were fixed in ethanol for staining with DNA dye and resuspended in 0.5 ml to 1 ml propidium iodide (PI) solution containing RNase and incubated at 37°C for 30 minutes. Cell-cycle profiles and the fractionations of apoptotic cells were obtained with a FACSvantage flow cytometer (Becton Dickinson, San Jose, CA, USA) and data were analyzed with ModFit software (Verity Software House, USA).

### RNA Isolation and Reverse Transcription*–*Polymerase Chain Reaction

Total cellular RNA was isolated using Trizol solution (Life Technologies, Grand Island, NY). By the method described elsewhere [8], reverse transcription (RT) was performed on RNA samples, followed by polymerase chain reaction (PCR) with the primers (Table 1) for STAT3, c-Myc, cyclin-D1 and survivin [16]–[19]. The PCR products were resolved on 1% agarose gel containing ethidium bromide (0.5 µg/ml), visualized and photographed using UVP Biospectrum Imaging System (UVP, Inc, Upland, CA). The β-actin PCR products generated from the same RT solution were cited as quantitative controls.

**Table 1:** Sequences of RT-PCR Primers.

Parameters	Primer Sequences	Annealing Temperature (°C)	Product Size (bp)	Reference
STAT3	F: 5′-gggtggagaaggacatcagcggtaa-3′	58	198	[16]
	R: 5′-gccgacaatactttccgaatgc-3′			
survivin	F: 5′-ggcatgggtgccccgacgttg-3′	58	439	[17]
	R: 5′-cagaggcctcaatccatggca-3′			
c-Myc	F: 5′-tggtcttcccctaccctctcaac-3′	56	265	[18]
	R: 5′-gatccagactctgaccttttgcc-3′			
cyclin D1	F: 5′-ctgtgctgcgaagtggaaaccat-3′	57	257	[19]
	R: 5′-ttcatggccagcgggaagacctc-3′			

### Immunocytochemical and Immunofluorescence Staining

Immunocytochemical staining (ICC) was performed on the coverslips obtained from each of the experimental groups by the method described elsewhere [20]. The antibodies against human STAT3, p-STAT3, survivin, cyclinD1, c-Myc, VEGF, Sirt1 and p53 were purchased from Santa Cruz Biotechnology, Inc, CA. Color reaction was developed using 3, 3′-diaminobenzidine tetrahydrochloride (DAB). According to the labeling intensity, the staining results were evaluated by two independent researchers and scored as negative (−) if no immunolabeling was observed in target cells, weakly positive (+) if the labeling was faint, moderately positive (++), and strongly positive (>++) when the labeling was stronger or distinctly stronger than (++). For immunofluorescence staining (IF), the cell bearing coverslips were rinsed with phosphate-buffered solution (PBS; pH 7.4) for 3 times, blocked with 10% goat serum in PBS (pH 7.4) for 20 minutes, incubated overnight with primary antibody against target protein and finally co-incubated with fluorescence-labeled goat anti-rabbit or rabbit anti-mouse IgG (1∶200; Santa Cruz Biotechnology, Inc) at 37°C for 60 minutes in darkness. The nuclei were labeled by DAPI (blue fluorescence). The coverslips were observed and photographed under a fluorescence microscope (BX53F, Olympus, Japan).

### Protein Preparation and Western Blotting

Total cellular proteins were prepared from the cells under different culture conditions [20]. The sample proteins (50 µg/well) were separated in 10% sodium dodecylsulfate-polyacrylamide gel electrophoresis and transferred to polyvinylidene difluoride membrane (Amersham, Buckinghamshire, UK). The membrane was blocked with 5% skimmed milk in TBS-T (10 mM Tris-HCl, pH8.0, 150 mM NaCl and 0.5% Tween 20) at 4°C, rinsed 10 minutes for three times with TBS-T, followed by 3 h incubation at room temperature with the first antibody and then 1 h incubation with HRP-conjugated anti-mouse or anti-rabbit IgG (Zymed Lab, Inc). The bound antibody was detected using the enhanced chemiluminescence system (Roche GmbH, Mannheim, Germany). After removing the labeling signal by incubation with stripping buffer [8], the membrane was reprobed with other antibodies one by one until all of the parameters were examined.

### Inhibition of STAT3 Activation with AG490

JAK2-specific inhibitor AG490 (Sigma) [21] was dissolved in DMSO and diluted to the final concentrations of 50 µM and 80 µM with culture medium. Four experimental groups were set as follows: Group1, normal culture; Group 2, treatment with 1.2‰ DMSO as background control; Groups 3 and 4, treatment with 50 µM or 80 µM AG490. The effects of AG490 on cell proliferation were determined by MTT, flow cytometry and coverslip-based morphological and ICC staining for STAT3, p-STAT3, cyclinD1, survivin, c-Myc and VEGF. RNA and protein samples were prepared from all groups for RT-PCR and Western blot analyses.

### Ethics Statement

Prior to the animal experiments, the research protocols were reviewed and approved by Animal Care and Use Committee of Dalian Medical University. All work involving experimental animals was performed in full compliance with NIH (National Institutes of Health) Guidelines for the Care and Use of Laboratory Animals. All experiments were performed under chloral hydrate anesthesia and all efforts were made to minimize suffering.

### Orthotopic TCC Model and Intravesical Treatment

Female BALB/c-nude mice (4 weeks, 14–16 g body weight) were raised under specific pathogen free (SPF) conditions. For establishing orthotopic bladder cancer model [22], mice were anesthetized intraperitoneally with 3.5% chloral hydrate (0.1 ml/10 g mice body weight) [23]. The state of anaesthesia was evaluated by loss of righting reflex (LORR), and time to induce LORR was usually between 5–10 minutes. In the germfree isolator, the mice urinary bladders were exposed through a low midline incision; 20 µl EJ cell suspension (1×10^6^ cells) was injected into its sub-epithelial layer and the appearance of “semitransparent bubbles” indicated successful inoculation. Three days later, the mice were randomly divided into two groups (10 mice/group) and treated by 50 µl 200 µM resveratrol or 50 µl 0.9% NaCl in two-day intervals. After urine evacuation, a 24-gauge Teflon-coated catheter was introduced into the lumen of the bladder. To avoid liquid leaking, the catheter attached with syringe was held in place for 40 minutes. All of the mice were anesthetized safely and usually recovered from anesthesia after 2 hours. By the end of 28 day experiment, the whole bladders were harvested, weighed and then subjected to histological and immunohistochemical (IHC) examinations. Apoptosis was detected by the TUNEL assay and its indexes were obtained by counting TUNEL-positive cells versus over 1000 observed cells in the tumor sections [24].

### Evaluation of Local Irritation and Systemic Toxicity

5-week old ICR female mice (5 mice/group) were intravesically treated with 50 µl of 200 µM resveratrol. The treatment was performed for 6 times in 2-day intervals. The mice treated with 50 µl of 2 mg/ml MMC (Sigma-Aldrich, dissolved in 0.9% NaCl) [25] in the same administration manner were cited as control. By the end of the treatments, the body weights were counted and the response(s) of bladder tissues to the treatments were examined in terms of mucosal integrity, capillary congestion and hemorrhage.

### Statistical Analysis

The differences in continuous variables were assessed by Student *t* test or one-way ANOVA. Statistical significance was defined as *P*<0.05.

## Results

### Short-term Resveratrol Treatment Suppressed TCC Cell Growth

MTT assay revealed that EJ cell growth was suppressed by short-term resveratrol treatments in dose- and time-related fashions (Figure 1B). H/E staining and TUNEL assay showed frequent apoptotic death in 2 h 150 µM and 200 µM Res-treated groups (Figure 1C). Flow cytometry showed that 2 h 150 µM and 200 µM Res treatments caused S-phase arrest and apoptosis in EJ cell populations. As shown in Figure 1D, the G1 and S fractions of EJ cells were 62.6% and 33% in normally cultured cells, which changed to 23.9% and 76.1% in 2 h 150 µM Res treated and 17.7% and 82.3% in 2 h 200 µM Res treated populations, respectively. The percentages of apoptosis in normally cultured, 2 h 150 µM Res- and 200 µM Res-treated groups were 0.394%, 4.98% and 11.4% at 72 h time point (Figure 1D). The growth of EJ cells was effectively inhibited by the constant treatment of 100 µM resveratrol.

### Resveratrol Inhibited STAT3 Transcription and Activation

**Figure 2:**
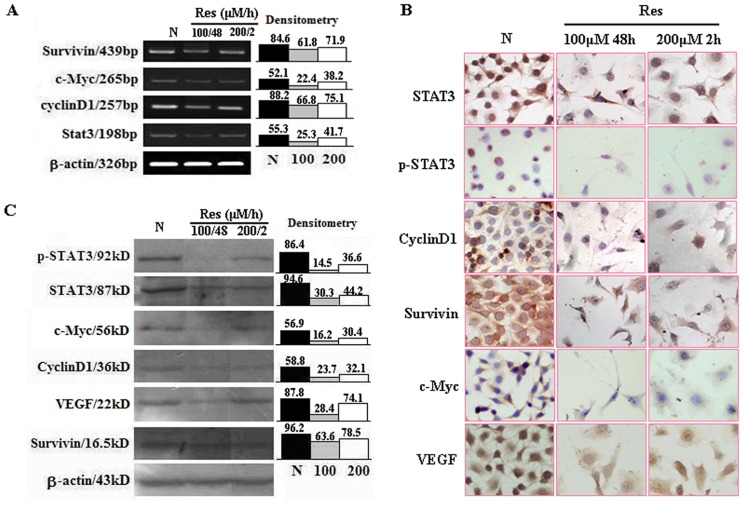
Evaluation of the levels of STAT3, p-STAT3, cyclinD1, survivin, c-Myc and VEGF in normally cultured EJ cells and the cells treated by resveratrol shortly (200 µM for 2 h) or constantly (100 µM for 48 h) by RT-PCR (A), immunocytochemical staining (B) and Western blotting (C).

Because of the critical roles of activated STAT3 signaling in bladder TCC cells [26], the effect of resveratrol on STAT3 signaling in EJ cells was analyzed by RT-PCR using the RNA samples extracted from the EJ cells without and with Res treatment. As shown in Figure 2A, STAT3 was expressed in normally cultured EJ cells and down-regulated after 2 h 200 µM Res treatment for 72 hours. ICC staining performed on the same experimental groups revealed strong STAT3 staining (>++) in either cytosolic space or nuclei of normally cultured EJ cells, which apparently weakened (+) after 72 hours of 2 h 200 µM Res treatment (Figure 2B; Table 2). It was also found that p-STAT3 was mainly localized in the nuclei of EJ cells and was diminished after 2 h 200 µM Res treatment for 72 hours (Figure 2B; Table 2). In accordance with the results of RT-PCR and ICC, Western blotting showed that 2 h 200 µM Res treatment caused distinct STAT3 and p-STAT3 reduction (Figure 2C). To further elucidate the effects of 2 h Res treatment on STAT3 signaling, the levels of STAT3 downstream genes (c-Myc, survivin, cyclinD1 and VEGF) in normally cultured and 2 h 200 µM Res treated EJ cells were examined by the methods described above. As shown in Figure 2, the expression of c-Myc, survivin, cyclinD1 and VEGF was suppressed in 2 h 200 µM Res-treated EJ cells (Table 2). Similar findings could also be detected in EJ cells treated constantly by 100 µM resveratrol for 48 h.

**Table 2:** Statuses of STAT3, p-STAT3 and STAT3 downstream genes in EJ cells and EJ transplanted tumors under different experimental conditions.

	EJ cells				Transplanted tumors	
	N	200 µMRes*	AG490		NaCl*	200 µM Res*
			50 µM	80 µM		
STAT3	+++	+	++	+	+++	++
p-STAT3#	++	−	−	−	++	−
cyclinD1	++	+	−	−	+++	++
Survivin	++	+	+	−	+++	++
c-Myc	++	+	+	+	+++	+
VEGF	+++	++	+	+	+++	+

*EJ cells treated by 2 h 200 µM Res in one day intervals for 3 times and the transplanted tumors treated by 2 h 200 µM Res or 0.9% NaCl in two-day intervals for 13 times.

# Densities of nuclear immuno-labeling.

### Inhibitory Effects of AG490 on EJ Cells

**Figure 3:**
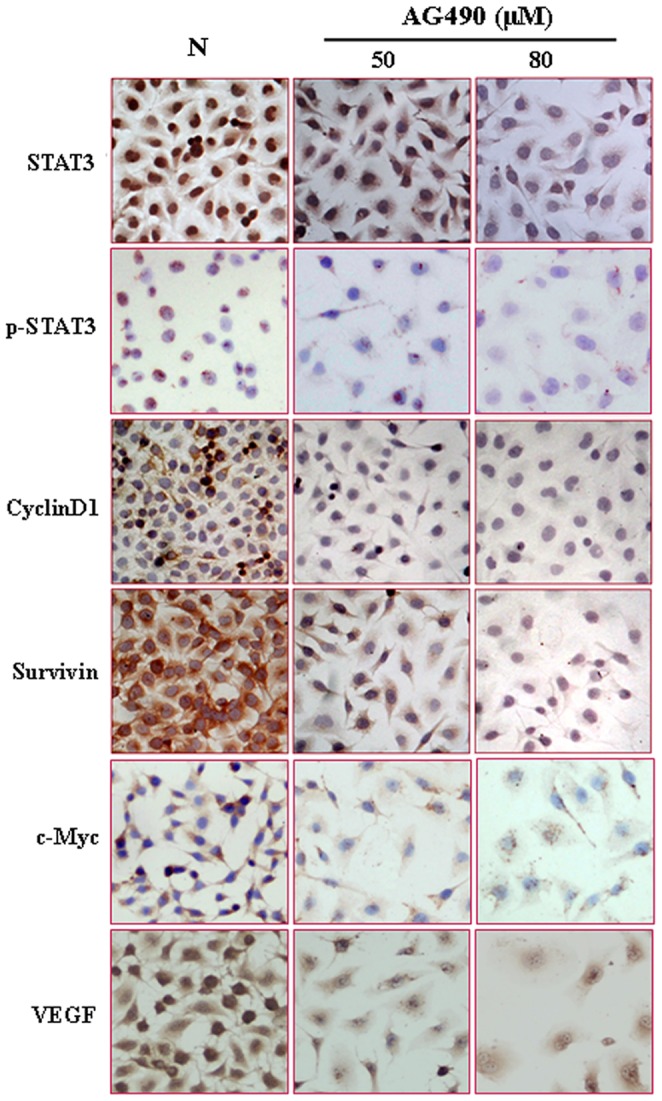
Immunocytochemical evaluation of the expression and intracellular distribution of STAT3, p-STAT3, cyclinD1, survivin, c-Myc and VEGF in EJ cells without and with AG490 treatments.

**Figure 4:**
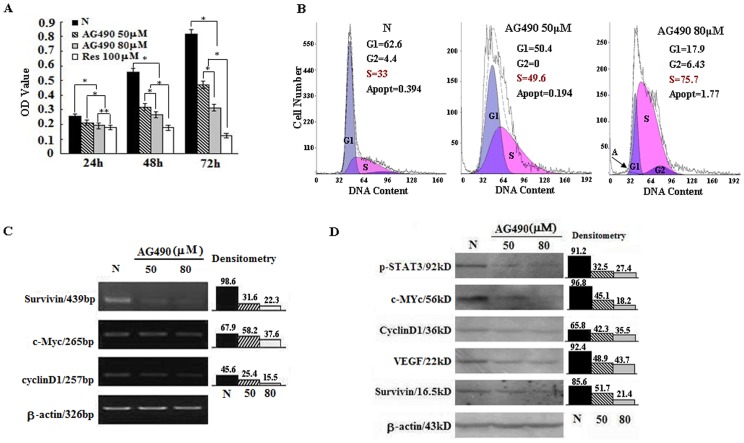
AG490 inhibited growth of EJ bladder cancer cells. A. MTT cell proliferation assay performed on normally cultured EJ cells and the cells with AG490 (50 µM and 80 µM) and constant 100 µM resveratrol treatments. *, *P*<0.01; **, *P*<0.05. B. Flow cytometry determination of cell cycle distribution and apoptosis in AG490 treated EJ cell population. C and D. Evaluation of p-STAT3, cyclinD1, survivin, c-Myc and VEGF levels in EJ cells without and with 50 µM or 80 µM AG490 treatments by RT-PCR and Western blot analyses.

To determine the significance of resveratrol-caused STAT3 inhibition, AG490, a selective inhibitor of STAT3 phosphorylation, was used to treat EJ cells [21]. Immunocytochemical staining demonstrated that STAT3 phosphorylation was inhibited in AG490-treated cells in terms of reduction of STAT3 and p-STAT3 nuclear labeling (Figure 3; Table 2). The proliferation of EJ cells was suppressed by AG490 in dose- and time-related fashion (Figure 4A). Flow cytometry analysis demonstrated that 48 hour 50 µM and 80 µM AG490 treatments caused S-phase arrest without inducing distinct apoptosis. The G1 and S fractions were 62.6% and 33% in normally cultured, 50.4% and 49.6% in 50 µM AG490 treated and 17.9% and 75.7% in 80 µM AG490 treated EJ cells (Figure 4B). These results are in consistence with those of short-term resveratrol treatments. The percentages of apoptosis in normally cultured, 50 µM AG490- and 80 µM AG490-treated groups at 48 h time point were 0.394%, 0.194% and 1.77%, respectively (Figure 4B). In comparison with normally cultured EJ cells, c-Myc, cyclinD1, survivin and VEGF expressions were down-regulated in AG490-treated cells (Figure 3, Figure 4C and D; Table 2).

### Intravesical Resveratrol Treatment Inhibited Orthotopic Tumor Growth

**Figure 5:**
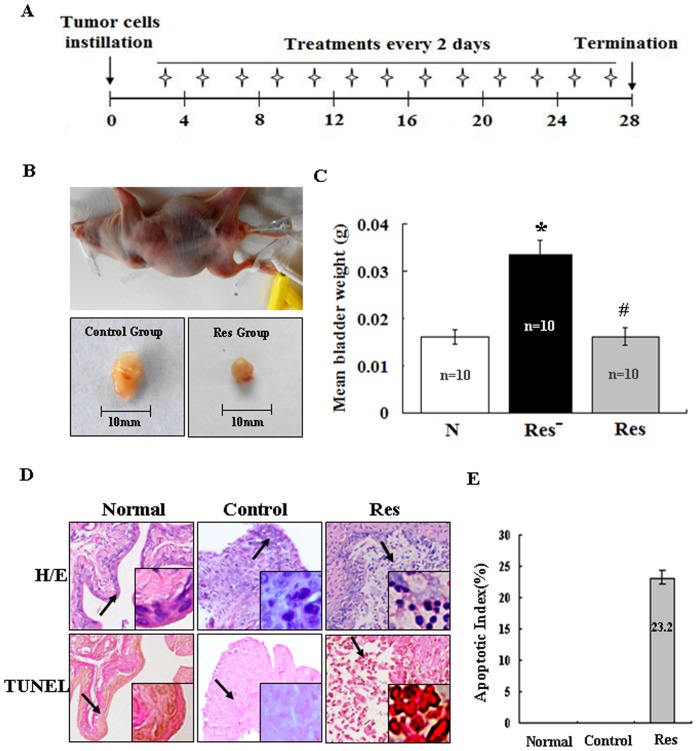
Growth inhibition and apoptosis induction of mouse orthotopic bladder tumors by 2 µM resveratrol intravesical instillation for 13 times in 2 day intervals. A. Schematic diagram of study design and dosing schedule. Treatments began 3 days after implantation (asterisks). B. Upper: Urethral catheterization of nude mice; Lower: the sizes of a pair of tumor-bearing urinary bladders without and with 2 h 200 µM resveratrol intravesical instillation for 13 times. C. Comparison of tumor burdens between resveratrol instillation group and the untreated control at Day 28 after tumor implantation. Normal nude mice (the same weeks old) bladders free of tumor implantation as control. *, compared with N group or Res group, *P*<0.01; #, compared with N group, *P*>0.05. D. H&E staining and TUNEL assay (the insets) for histological and apoptosis evaluation of urinary bladder tissues with intravesical resveratrol treatment (2 h 200 µM resveratrol for 13 times in 2 day intervals). Normal: the bladder of a nude mouse; Control: the bladder bearing orthotopic transplanted tumor without resveratrol treatment (0.9% NaCl treatment); Res: the bladder bearing orthotopic transplanted tumor with 200 µM resveratrol treatment for 2 h and 13 times. E. Evaluation of apoptotic indexes among Normal, Control and Res groups.

**Figure 6:**
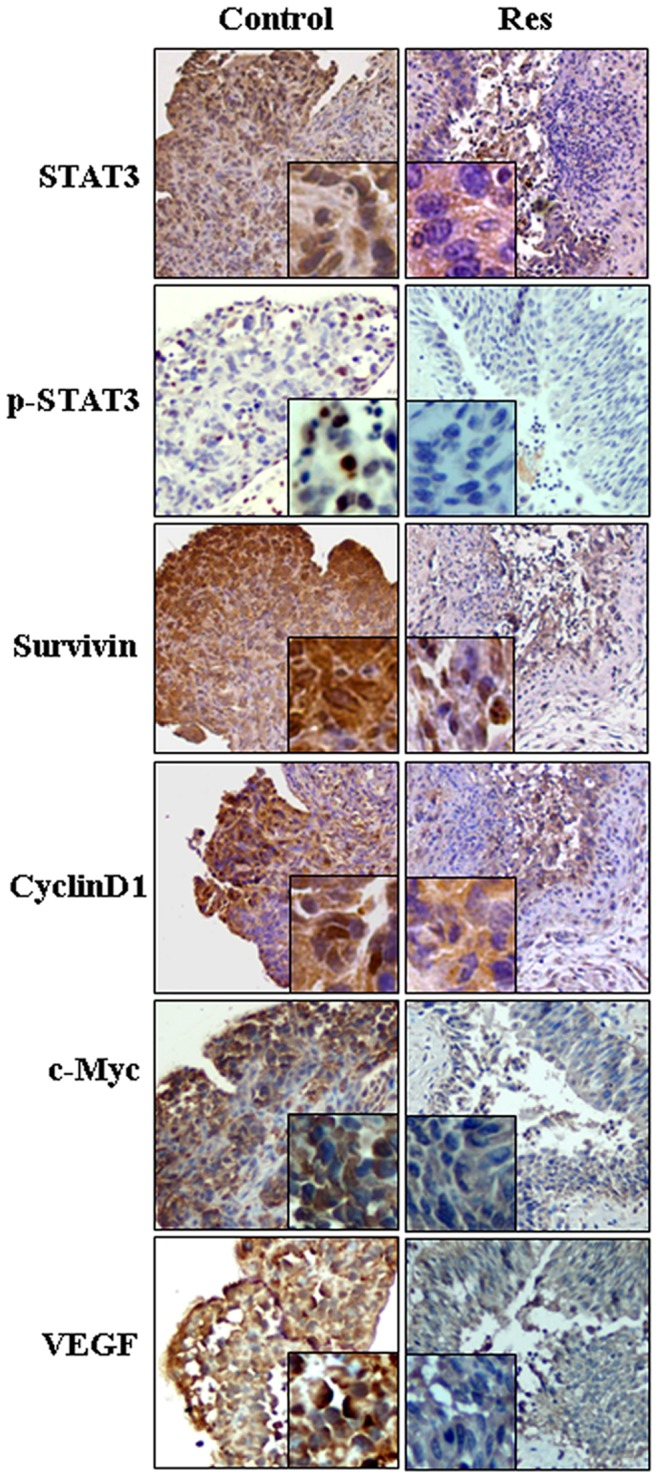
Inhibited STAT3 activation in mouse orthotopic bladder tumors treated by intravesical resveratrol instillation. H&E and immunohistochemical stainings of STAT3, p-STAT3, cyclinD1, survivin, c-Myc and VEGF in the bladder tissue samples treated by 0.9% NaCl (control) or 200 µM resveratrol (Res) for two hours and 13 times. Insets, the enlarged images of tumor tissues.

H/E staining was performed on the inoculated sites and confirmed that the successful rate of orthotopic tumor implantation was 100% (20/20). The schematic diagram of intravesical resveratrol treatment and dosing schedule was shown in Figure 5A. The tumor burdens were determined by weighing the whole bladders harvested from all groups [27], which revealed the greater mean bladder weight in NaCl-treated control group than that in resveratrol-treated group (0.03362 g vs 0.01625 g, *P*<0.01; Figure 5B and C). TUNEL assay performed on orthotopic tumor samples with and without short-term resveratrol instillation demonstrated remarkable apoptotic death (apoptotic index = 23.2%) in the former cases (Figure 5D and E). Immunohistochemical staining showed that STAT3, p-STAT3 and its downstream genes c-Myc, cyclinD1, survivin and VEGF could be detected in either cytoplasm or the nuclei of tumor tissues in NaCl control group but became apparently reduced in resveratrol-treated tumors (Figure 6; Table 2).

### Short-term Resveratrol Treatment Promoted the Nuclear Translocation of Sirt1 and p53

**Figure 7:**
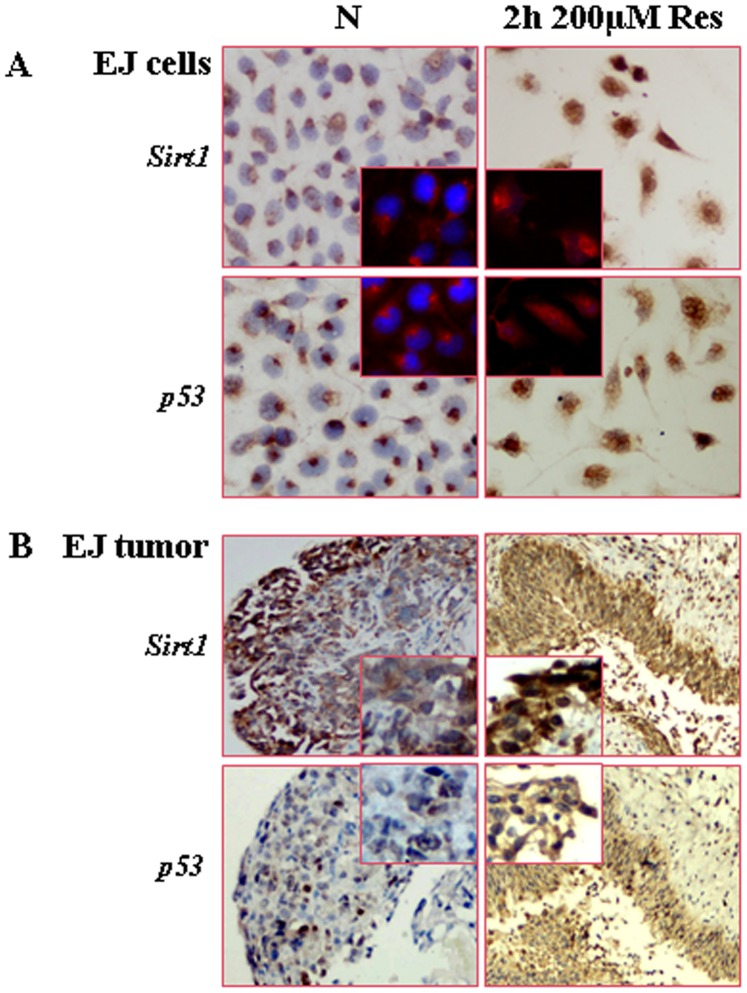
Short-term resveratrol treatment promoted nuclear translocations of Sirt1 and p53. A. Evaluation of Sirt1 and p53 distribution in normally cultured EJ cells and the cells treated by 2 µM resveratrol at 72 h time-point by immunocytochemical and immunofluorescence stainings (the insets). B. Sirt1 and p53 oriented immunohistochemical staining of the bladder tissues treated by 0.9% NaCl (control) or 200 µM resveratrol (Res) for two hours and 13 times. Insets, the enlarged images of tumor tissues.

Resveratrol is known as an activator of silent mating type information regulation 2 homolog (Sirt1) that modulates p53 activity by deacetylation [28], [29]. Therefore, the in vitro and in vivo influences of resveratrol on Sirt1 activity of EJ cells were investigated by the methods of ICC, IHC and IF. As shown in Figure 7, both Sirt1 and p53 were distributed in cytosolic space of EJ cells and transplanted tumors without resveratrol treatment, which were apparently translocated into the nuclei of EJ cells after in vitro 2 h 200 µM Res treatment at 72 h time-point and 2 h intravesical resveratrol instillation for 13 times, suggesting potential functional link of these two proteins.

### No Obvious Local Irritation of Resveratrol-treated Bladder Mucosa

**Figure 8:**
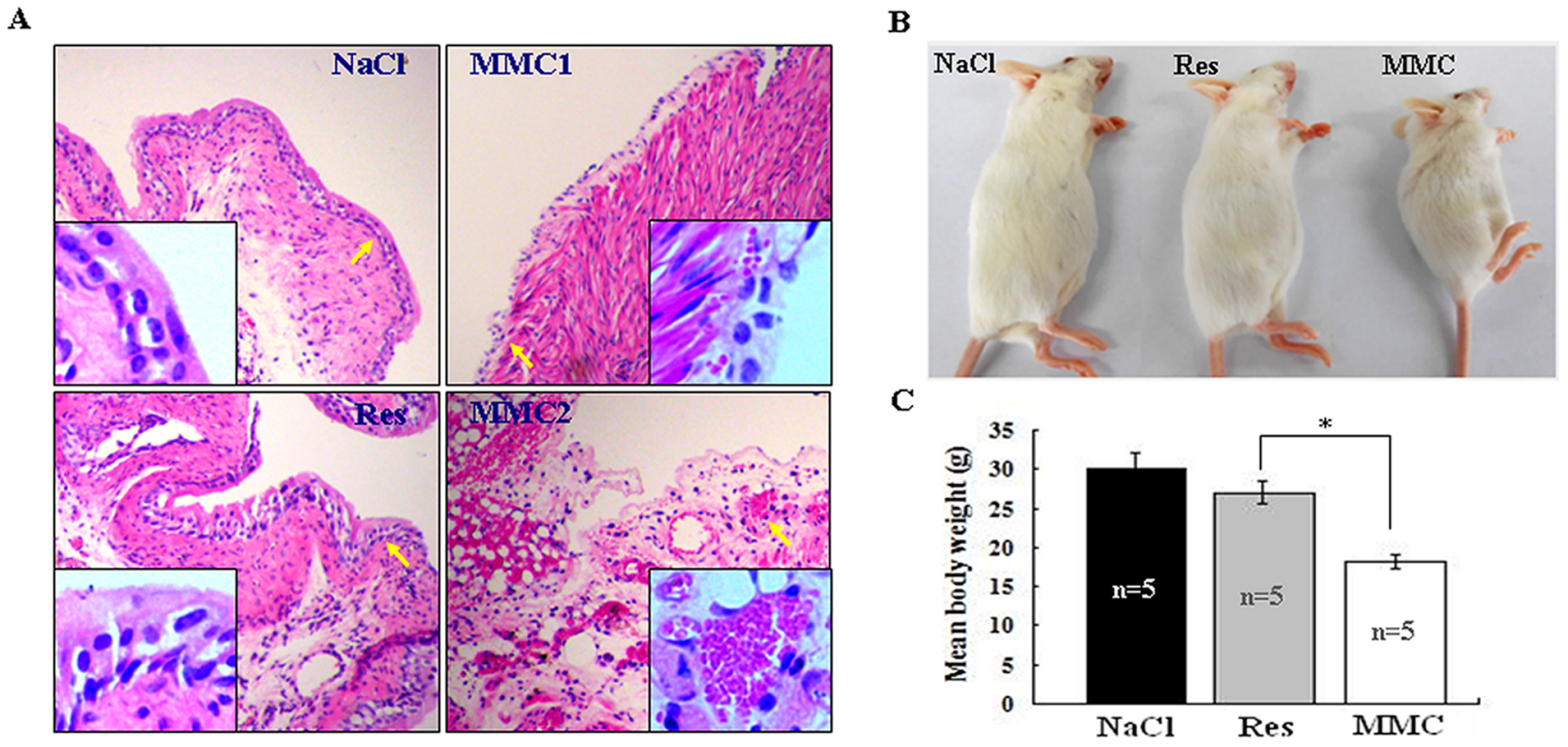
Comparison of bladder irritation (A) and the body weights (B and C) of the mice treated by resveratrol and mitomycin C (MMC) intravesical instillation. NaCl, ICR mice treated by intravesical instillation of 0.9% NaCl; Res, 200 µM resveratrol; MMC, 2 mg/ml mitomycine C. These treatments were conducted for 6 times in 2-day intervals. Severe epithelial damage, capillary congestion and hemorrhage could be observed in the MMC-treated but not in NaCl and Res-treated bladders. Insets, high magnification images of arrow-indicated urothelial regions. *P<0.01.

Intravesical resveratrol instillation was conducted on both female tumor-free ICR mice and the female tumor-bearing nude mice. By the end of the experiment, the whole bladders were removed and fixed in 10% neutral buffered formalin for H/E staining-based cystitis examination. As shown in Figure 8A, the transitional epithelia of urinary bladder walls were intact, and neither distinct capillary congestion nor inflammatory lymphocyte infiltration was observed in the sub-mucosal space of resveratrol-treated cancer-free ICR mice. Similar situation was also found in the tumor surrounding bladder tissues of tumor-bearing nude mice treated by intravesical resveratrol instillation (data not shown). In contrast, severe epithelial damage, capillary congestion and hemorrhage could be observed in the MMC-treated urinary bladders of ICR mice (Figure 8A). The distinct body weight loss was found in MMC-treated group (18.1 g in average) in comparison with that in Res-treated group (27.2 g in average; *P*<0.01; Figure 8B and 8C).

## Discussion

Transitional cell carcinoma of urinary bladder/TCC is responsible for over 130 000 annual deaths worldwide [30] largely due to the recurrence and metastasis [2]. Intravesical administration of anticancer agents has been adopted to eradicate residual tumor cells [3], [4]. However, those conventional therapies can not last long because of systemic toxicity and local irritation [2]. Since resveratrol is non-toxic [5] and exerts inhibitory effects on TCC cells [9]–[12], it would be an alternative candidate for the management of TCCs if its anti-TCC capacity can be further proved under the experimental conditions closer to the clinical statuses. To mimic intravesical drug instillation, the cultured EJ TCC cells were treated with 100 µM, 150 µM or 200 µM resveratrol transiently instead of constantly. The results clearly demonstrated that 150 µM and 200 µM resveratrol treatments for 1.5-hour or 2-hour were sufficient to suppress growth and induce apoptosis of EJ cells, suggesting that short-term resveratrol treatment could achieve similar anti-TCC effects as that of conventional drugs [14]. This notion was further supported by the downregulation of STAT3 and STAT3 target gene expression and reduction of p-STAT3 nuclear translocation in resveratrol-treated EJ cells, because activated STAT3 signaling was associated with TCC cell growth and survival [26]. These promising in vitro results encourage us to testify whether short-term resveratrol instillation can also lead to similar therapeutic consequence in an appropriate orthotopic xenograft model.

Although anti-cancer effects of resveratrol have been evidenced, this agent has not yet been applied to clinical practice largely due to the low intracellular bioavailability of systemically administrated resveratrol [31], [32]. Unlike solid tumors in majority of organs, TCCs grow outward to the bladder cavity. Moreover, the instilled drug can be easily retained in the bladder and directly exerts its effects on the tumor tissue/cells. Therefore, an orthotopic xenograft model was established in nude mouse urinary bladder, which was then treated by resveratrol in the same dosages as that of the in vitro experiment and in the manner similar with clinical practice [14]. It was found that the tumor growth was remarkably suppressed and tumor cells underwent extensive apoptosis. Since the experiment was performed on the tumors inside the bladder, the results obtained were closer to the patients’ reality and, therefore, would be of translational values.

One of the severe side-effects of intravesical chemotherapy with conventional anticancer drugs is hemorrhagic cystitis [33]. Although the non-toxicity of resveratrol to normal cells has been described in some organs [32], the effect(s) of resveratrol on normal urinary bladder tissue remains unknown. Therefore, an effective anti-TCC dose of resveratrol (200 µM) was instilled into the bladders of cancer-free mice and compared with that treated by 2 mg/ml mitomycin C. Meanwhile, the response(s) of transplanted tumors and their surrounding mucosa to resveratrol were compared. We found that both the cancer-free and the tumor-surrounding mucosa showed almost intact transitional epithelia without distinct capillary congestion and inflammatory lymphocyte infiltration, while severe irritation and epithelial damage happened to the mitomycin C-treated mucosa. Our data thus provide in vivo evidence of cancer-targeting property of resveratrol in the urinary bladder and further suggest the suitability of resveratrol in clinical treatment of human TCCs.

Resveratrol possesses multifaceted molecular effects including inhibition of STAT3 signaling [20] and activation of Sirt1 deacetylation [28]. STAT3 activation is frequent in TCCs and its inhibition may lead to growth arrest and apoptosis [26]. We found that STAT3 signaling is also activated in EJ TCC cells and short term in vitro and in vivo resveratrol treatments can inhibit STAT3 activation and down-regulate the expression of STAT3 and its downstream cancer-associated genes. These results together with the similar findings from other types of cancers [20], [32], [34] suggest that STAT3 is a major molecular target of resveratrol. The suppressed growth of AG490-treated EJ cells further supports the critical roles of activated STAT3 signaling in promoting TCC cell proliferation. The lack of apoptotic death in AG490-treated EJ population indicates that resveratrol may alter other cell survival machineries beyond STAT3 signaling. So far, the role of Sirt1 in TCC formation and progression remains unclear. In this study, Sirt1 nuclear translocation is evidenced in resveratrol-treated EJ cells, paralleled with p53 nuclear translocation. It has been reported that Sirt1 nucleocytoplasmic shuttling plays a critical role in the regulation of Sirt1 activity [35]. Moreover, activated Sirt1-p53 pathway can induce senescence-like growth arrest of cancer cells [36]. Our results thus provide a cue to explore additional anti-TCC mechanism of resveratrol and to elucidate the biological implications of nuclear co-translocation of Sirt1 and p53 proteins.

In conclusions, the safety and efficacy of resveratrol in clinical treatment of bladder TCCs have been validated here by shortly exposing the cultured human TCC EJ cells and orthotopic transplanted tumors to resveratrol. Two hours 200 µM resveratrol treatment is sufficient to suppress in vitro and in vivo growth and to induce distinct apoptosis of EJ cells, presumably through inhibition of STAT3 activation and induction of Sirt1 and p53 nuclear translocation. Moreover, resveratrol neither exerts harmful effects on normal urothelial cells nor causes bladder hemorrhagic cystitis and body weight loss. Our results obtained from the clinically relevant experimental models strongly suggest the practical values of resveratrol in the management of human TCCs.

## References

[1] Cheung G, Sahai A, Billlia M, Dasgupta P, Khan MS (2013) Recent advances in the diagnosis and treatment of bladder cancer. BMC Med 11: 13. 23327481 10.1186/1741-7015-11-13 PMC3566975

[2] Houghton BB, Chalasani V, Hayne D, Grimison P, Brown CS, et al (2013) Intravesical chemotherapy plus bacille Calmette-Guérin in non-muscle invasive bladder cancer: a systematic review with meta-analysis. BJU Int 111: 977–983. 23253618 10.1111/j.1464-410X.2012.11390.x

[3] Pinto-Leite R, Arantes-Rodrigues R, Palmeira C, Colaço B, Lopes C, et al (2013) Everolimus combined with cisplatin has a potential role in treatment of urothelial bladder cancer. Biomed Pharmacother 67: 116–121. 23433853 10.1016/j.biopha.2012.11.007

[4] Barlow LJ, Benson MC (2013) Experience with newer intravesical chemotherapy for high-risk non-muscle-invasive bladder cancer. Curr Urol Rep 14: 65–70. 23378162 10.1007/s11934-013-0312-2

[5] Chen HJ, Hsu LS, Shia YT, Lin MW, Lin CM (2012) The β-catenin/TCF complex as a novel target of resveratrol in the Wnt/β-catenin signaling pathway. Biochem Pharmacol 84: 1143–1153. 22935447 10.1016/j.bcp.2012.08.011

[6] Yaseen A, Chen S, Hock S, Rosato R, Dent P, et al (2012) Resveratrol sensitizes acute myelogenous leukemia cells to histone deacetylase inhibitors through reactive oxygen species-mediated activation of the extrinsic apoptotic pathway. Mol Pharmacol 82: 1030–1041. 22923501 10.1124/mol.112.079624 PMC3502626

[7] Leon-Galicia I, Diaz-Chavez J, Garcia-Villa E, Uribe-Figueroa L, Hidalgo-Miranda A, et al (2013) Resveratrol induces downregulation of DNA repair genes in MCF-7 human breast cancer cells. Eur J Cancer Prev 22: 11–20. 22644231 10.1097/CEJ.0b013e328353edcb

[8] Wen S, Li H, Wu ML, Fan SH, Wang Q, et al (2011) Inhibition of NF-κB signaling commits resveratrol-treated medulloblastoma cells to apoptosis without neuronal differentiation. J Neuro-oncol 104: 169–177. 10.1007/s11060-010-0496-y 21161674

[9] Bai Y, Mao QQ, Qin J, Zheng XY, Wang YB, et al (2010) Resveratrol induces apoptosis and cell cycle arrest of human T24 bladder cancer cells in vitro and inhibits tumor growth in vivo. Cancer Sci 101: 488–493. 20028382 10.1111/j.1349-7006.2009.01415.x PMC11159480

[10] Lin X, Wu G, Huo WQ, Zhang Y, Jin FS (2012) Resveratrol induces apoptosis associated with mitochondrial dysfunction in bladder carcinoma cells. Int J Urol 19: 757–764. 22607368 10.1111/j.1442-2042.2012.03024.x

[11] Stocco B, Toledo K, Salvador M, Paulo M, Koyama N, et al (2012) Dose-dependent effect of resveratrol on bladder cancer cells: chemoprevention and oxidative stress. Maturitas 72: 72–78. 22386766 10.1016/j.maturitas.2012.02.004

[12] García Mediero JM, Ferruelo Alonso A, Páez Borda A, Luján Galán M, Angulo Cuesta J, et al (2005) Effect of polyphenols from the Mediterranean diet on proliferation and mediators of in vitro invasiveness of the MB-49 murine bladder cancer cell line. Actas Urol Esp 29: 743–749. 16304905 10.1016/s0210-4806(05)73335-0

[13] Parada LF, Tabin CJ, Shih C, Weinberg RA (1982) Human EJ bladder carcinoma oncogene is homologue of Harvey sarcoma virus ras gene. Nature 297: 474–478. 6283357 10.1038/297474a0

[14] Liu J, Wang Q, Wang X, Sun Y, Chen XY, et al (2003) Apoptosis of bladder cancer cells induced by short-term and low-dose Mitomycin-C: potential molecular mechanism and clinical implication. Int J Mol Med 11: 389–394. 12579346

[15] Colombo R, Rocchini L, Suardi N, Benigni F, Colciago G, et al (2012) Neoadjuvant short-term intensive intravesical mitomycin C regimen compared with weekly schedule for low-grade recurrent non-muscle-invasive bladder cancer: preliminary results of a randomised phase 2 study. Eur Urol 62: 797–802. 22633362 10.1016/j.eururo.2012.05.032

[16] Gibbs CP, Kukekov VG, Reith JD, Tchigrinova O, Suslov ON, et al (2005) Stem-like cells in bone sarcomas: implications for tumorigenesis. Neoplasia 7: 967–976. 16331882 10.1593/neo.05394 PMC1502023

[17] Ikeguchi M, Kaibara N (2001) Changes in survivin messenger RNA level during cisplatin treatment in gastric cancer. Int J Mol Med 8: 661–666. 11712083 10.3892/ijmm.8.6.661

[18] Grotzer MA, Hogarty MD, Janss AJ, Liu X, Zhao H, et al (2001) MYC messenger RNA expression predicts survival outcome in childhood primitive neuroectodermal tumor/medulloblastoma. Clin Cancer Res 7: 2425–2433. 11489822

[19] Simile MM, De Miglio MR, Muroni MR, Frau M, Asara G, et al (2004) Down-regulation of c-myc and Cyclin D1 genes by antisense oligodeoxy nucleotides inhibits the expression of E2F1 and in vitro growth of HepG2 and Morris 5123 liver cancer cells. Carcinogenesis 25: 333–341. 14604889 10.1093/carcin/bgh014

[20] Yu LJ, Wu ML, Li H, Chen XY, Wang Q, et al (2008) Inhibition of STAT3 expression and signaling in resveratrol-differentiated medulloblastoma cells. Neoplasia 10: 736–744. 18592012 10.1593/neo.08304 PMC2435009

[21] Cirone M, Di Renzo L, Lotti LV, Conte V, Trivedi P, et al (2012) Primary effusion lymphoma cell death induced by bortezomib and AG490 activates dendritic cells through CD91. PLoS ONE 7: e31732. 22412839 10.1371/journal.pone.0031732 PMC3296697

[22] Chan E, Patel A, Heston W, Larchian W (2009) Mouse orthotopic models for bladder cancer research. BJU Int 104: 1286–1291. 19388981 10.1111/j.1464-410X.2009.08577.x

[23] Vachon P, Faubert S, Blais D, Comtois A, Bienvenu JG (2000) A pathophysiological study of abdominal organs following intraperitoneal injections of chloral hydrate in rats: comparison between two anaesthesia protocols. Lab Anim 34: 84–90. 10759371 10.1258/002367700780578082

[24] Chen C, Zhou H, Liu X, Liu Z, Ma Q (2011) Reduced Expression of von Hippel-Lindau Protein Correlates with Decreased Apoptosis and High Chondrosarcoma Grade. J Bone Joint Surg Am 93: 1833–1840. 22005870 10.2106/JBJS.I.01553

[25] Hadaschik BA, Adomat H, Fazli L, Fradet Y, Andersen RJ, et al (2008) Intravesical chemotherapy of high-grade bladder cancer with HTI-286, a synthetic analogue of the marine sponge product hemiasterlin. Clin Cancer Res 14: 1510–1518. 18316576 10.1158/1078-0432.CCR-07-4475

[26] Chen CL, Cen L, Kohout J, Hutzen B, Chan C, et al (2008) Signal transducer and activator of transcription 3 activation is associated with bladder cancer cell growth and survival. Mol Cancer 7: 78. 18939995 10.1186/1476-4598-7-78 PMC2577686

[27] Chan ES, Patel AR, Hansel DE, Larchian WA, Heston WD (2012) Sunitinib malate provides activity against murine bladder tumor growth and invasion in a preclinical orthotopic model. Urology 80: 736.e1–736.e5. 10.1016/j.urology.2012.04.038 22676953

[28] Zou T, Yang Y, Xia F, Huang A, Gao X, et al (2013) Resveratrol Inhibits CD4+ T cell activation by enhancing the expression and activity of Sirt1. PLoS ONE 8: e75139. 24073240 10.1371/journal.pone.0075139 PMC3779207

[29] Hori YS, Kuno A, Hosoda R, Horio Y (2013) Regulation of FOXOs and p53 by SIRT1 modulators under oxidative stress. PLoS ONE 8: e73875. 24040102 10.1371/journal.pone.0073875 PMC3770600

[30] Ferlay J, Shin HR, Bray F, Forman D, Mathers C, et al (2010) Estimates of worldwide burden of cancer in 2008: GLOBOCAN 2008. Int J Cancer 127: 2893–2917. 21351269 10.1002/ijc.25516

[31] Shu XH, Li H, Sun Z, Wu ML, Ma JX, et al (2010) Identification of metabolic pattern and bioactive form of resveratrol in human medulloblastoma cells. Biochem Pharmacol 79: 1516–1525. 20105429 10.1016/j.bcp.2010.01.022

[32] Shu XH, Li H, Sun XX, Wang Q, Sun Z, et al (2011) Metabolic patterns and biotransformation activities of resveratrol in human glioblastoma cells: relevance with therapeutic efficacies. PLoS ONE 6: e27484. 22096581 10.1371/journal.pone.0027484 PMC3214056

[33] Abraham P, Isaac B, Ramamoorthy H, Natarajan K (2011) Oral glutamine attenuates cyclophosphamide-induced oxidative stress in the bladder but does not prevent hemorrhagic cystitis in rats. J Med Toxicol 27: 118–124. 10.1007/s13181-010-0103-9 PMC3724431 20661687

[34] Quoc Trung L, Espinoza JL, Takami A, Nakao S (2013) Resveratrol induces cell cycle arrest and apoptosis in malignant NK cells via JAK2/STAT3 pathway inhibition. PLoS ONE 8: e55183. 23372833 10.1371/journal.pone.0055183 PMC3555980

[35] Tong C, Morrison A, Mattison S, Qian S, Bryniarski M, et al (2013) Impaired SIRT1 nucleocytoplasmic shuttling in the senescent heart during ischemic stress. FASEB J 27: 4332–4342. 23024374 10.1096/fj.12-216473 PMC3804750

[36] Takwi A, Li Y (2009) The p53 pathway encounters the microRNA world. Curr Genomics 10: 194–197. 19881912 10.2174/138920209788185270 PMC2705852

